# Sherlock—A Free and Open-Source System for the Computer-Assisted Structure Elucidation of Organic Compounds from NMR Data

**DOI:** 10.3390/molecules28031448

**Published:** 2023-02-02

**Authors:** Michael Wenk, Jean-Marc Nuzillard, Christoph Steinbeck

**Affiliations:** 1 Institute for Inorganic and Analytical Chemistry, Friedrich-Schiller-University, 07743 Jena, Germany; 2 CNRS, Université de Reims Champagne-Ardenne, ICMR, 51097 Reims, France

**Keywords:** natural products, nuclear magnetic resonance, dereplication, structure elucidation, databases, open-source, NMRium, pyLSD, nmrXiv

## Abstract

The structure elucidation of small organic molecules (<1500 Dalton) through 1D and 2D nuclear magnetic resonance (NMR) data analysis is a potentially challenging, combinatorial problem. This publication presents Sherlock, a free and open-source Computer-Assisted Structure Elucidation (CASE) software where the user controls the chain of elementary operations through a versatile graphical user interface, including spectral peak picking, addition of automatically or user-defined structure constraints, structure generation, ranking and display of the solutions. A set of forty-five compounds was selected in order to illustrate the new possibilities offered to organic chemists by Sherlock for improving the reliability and traceability of structure elucidation results.

## 1. Introduction

NMR spectroscopy is the most widely used analytical method for the thorough identification of organic chemical compounds. Preliminarily to *de novo* structure elucidation by NMR, database lookup for an already known compound (dereplication) or for structure fragments has been included in several existing CASE systems using the spectral fingerprint of one-dimensional (1D) NMR experiments as a search key. Through-bond proximity relationships between atoms became available with the possibility of routinely recording two-dimensional (2D) NMR spectra. The typical collection of NMR data used for computer-assisted structure elucidation (CASE) might contain 1D spectra such as ^1^H, ^13^C and DEPT as well as the 2D HSQC, HMBC and COSY spectra. The HSQC experiment indicates direct bonds between heavy atoms (non-hydrogen) and protons whereas HMBC and COSY spectra bear hints about long-range chemical shift correlations between protons and heavy atoms (HMBC) or between protons (COSY). Together with a given molecular formula (MF) mainly determined by mass spectrometry (MS), the substructures and restrictions derived from 1D and 2D NMR spectra form the cornerstone of structure generation by CASE systems. These constraints and the ones resulting from fragment search shrink the chemical search space that would be otherwise too wide for practical applications [1,2,3,4,5,6,7,8].

Due to the combinatorial possibilities that might still arise in constitutional space, erroneous structural assignments were published, as mentioned in earlier reviews [2,4]. Several CASE programs have been reported to address this problem and to exhaustively generate all possible structures which satisfy structure restrictions given by automatic detection or user definition. Examples are COCON [9], SENECA [10], LSD/pyLSD [11,12], Schmarnica [13], Mestrelab MNova [14], Bruker CMC-se [14,15] and ACD/Structure Elucidator [16,17].

Until now and to the best of our knowledge, only commercial and closed-source software exist which include a frontend offering spectrum processing routines to obtain structural information from NMR data and a backend which provides a suite of CASE-related routines. 

To close this gap, we developed Sherlock, a CASE expert system for the easy identification of known organic compounds and, when necessary, for their *de novo* structure elucidation. 

## 2. Results and Discussion

### 2.1. The Test Dataset

The performance of Sherlock was evaluated with 45 test data sets which are available as freely accessible archives (see Section 5.2).

An overview of the structure search settings and results is given in Table 1. The system presented here was able to handle and solve problems with a number of heavy atoms up to 40 (test case 19). More is likely possible but was not evaluated. With the inclusion of the default settings, the generated lists of solutions contained the expected molecule in 37 of 45 cases. The last five problems (41 to 45) could not be solved within an acceptable time. There, the structure search was manually interrupted after three hours of computing time. This does not necessarily mean that the CASE system is not able to solve them. Further analysis by NMR experts might lead to results based on additional user-defined constraints.

The resolution parameters had to be adjusted in three examples (5, 17, and 25) to produce the expected structure. For example, the automatic determination to allow hetero-hetero bonds in study case 17 sets that property to false due to an occurrence of hetero-hetero atom bond lower than the threshold of 1%. Thus, the bond between the two nitrogen atoms was never formed. After allowing it manually the expected solution was generated. In another example, test case 25, for the carbon with a chemical shift of 131.4, the detection routine proposed an *sp^2^* hybridization and another carbon as a mandatory neighbour. Since it is supposed to be located between nitrogen and sulphur, a carbon as a direct neighbour was not appropriate here. Additionally, the list of possible hybridizations should include *sp*. Finally, to enable the generation of the desired molecule, the modification of the hybridization and set neighbours threshold to 0.1% and 100% was necessary.

After the three necessary parameter adjustments and the elucidation for each of the first 40 test examples, the rank of the expected compound was determined. As a result, 38 of 40 candidate lists contained the desired structure in the first place whereas two of them were ranked #6 and #8. This indicates that the prediction and ranking modules of Sherlock are reliable and the expected compound is often situated in the top ten of the ranked candidate list. A full spectrum-to-spectrum assignment (Table 1) was produced in 31 cases among 40. A 0.83 ppm overall mean average chemical shift deviation value was achieved.

The number of unassigned signals ranges from one to four in the 9 remaining cases. One reason why the prediction of signals might fail is that there is no entry in the HOSE code library and thus no prediction value is available. This problem will be overcome when larger open-access collections of assigned NMR spectra will become available. Another cause for a missing signal could be a larger difference in the given maximum shift tolerance or average deviation. Furthermore, diastereotopic carbons might not be distinguished by their 3D HOSE code since the stereochemical information in the output of pyLSD is not provided and no method in Sherlock is implemented to provide it. In addition, the required stereochemical properties, as suggested [18,19], are not available for all compounds in Sherlock’s structure-to-spectrum database which leads to the same problem (mentioned above) during the HOSE code knowledge base creation and thus also affects the prediction capability.

**Figure 1:**
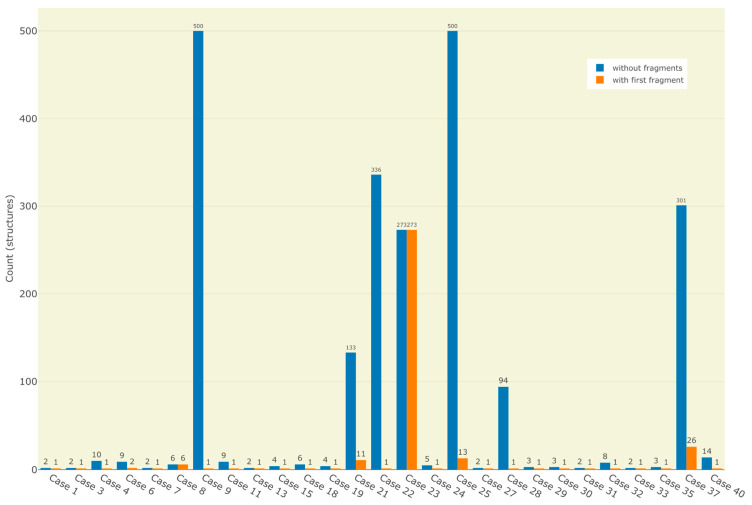
Distributions of number of results before and after the application of the first fragment during the structure generation process. Only test cases with multiple candidates before and at least one result after are displayed.

Sherlock provides the search for fragments for ^13^C query spectra. This was tested for the first 40 case studies as well, by incorporating the first entry of the ranked fragment list in the input file of the structure generator. In the vast majority of the cases (32) the number of candidates was equal to one and the solution was the expected one. Only six of the problems resulted in more than one solution but mostly with a massive reduction in solution size. In two cases (14 and 17) no structure was produced due to improper fragment proposal. Parameter adjustments for the fragment search in case of example 9, 30 and 37 led to proper first fragment suggestions and the production of results, including the expected one. The impact of the inclusion of the first proposed fragment in solution structures is illustrated in Figure 1.

**Figure 2:**
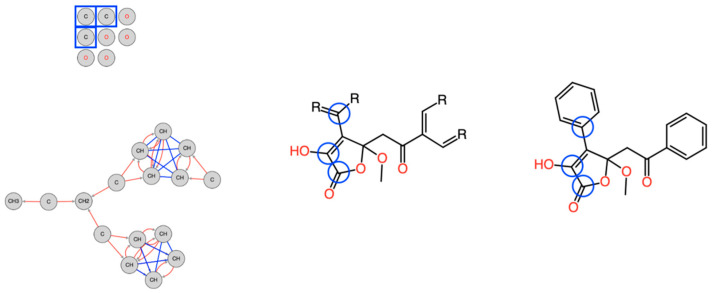
Molecular Connectivity Diagram (**left**), first fragment (**center**) and final result (**right**) of study case 22. The blue framed quaternary carbons had no identified correlation in the HMBC spectrum. Thus, the MCD contains no connection for those atoms to other atoms. Including the fragment in the elucidation process led to reducing the number of solutions from 336 to one.

For example, test case 22 shows a drastic reduction of the solution set size. From 336 solutions without fragment data, only one structure was left. This was due to the discovery of a fragment covering most of the structure, i.e., the quaternary carbon atoms for which no correlations could be identified in the HMBC spectrum (Figure 2). In addition, the connections of all hetero atoms (oxygens) are provided which otherwise would increase the search space enormously without any further structural information.

### 2.2. Test Case 15

**Figure 3:**
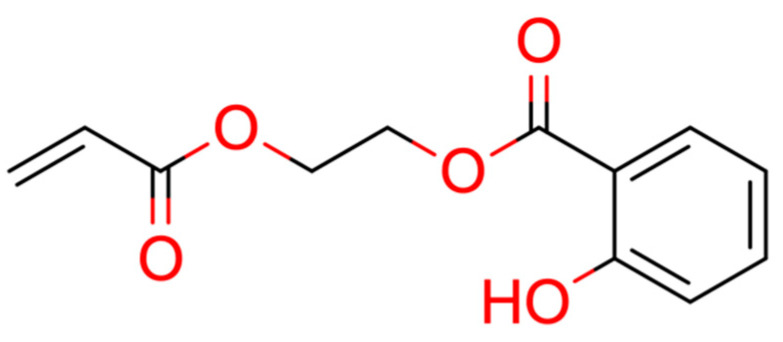
Structure of test compound 15 (2-prop-2-enoyloxyethyl 2-hydroxybenzoate).

**Figure 4:**
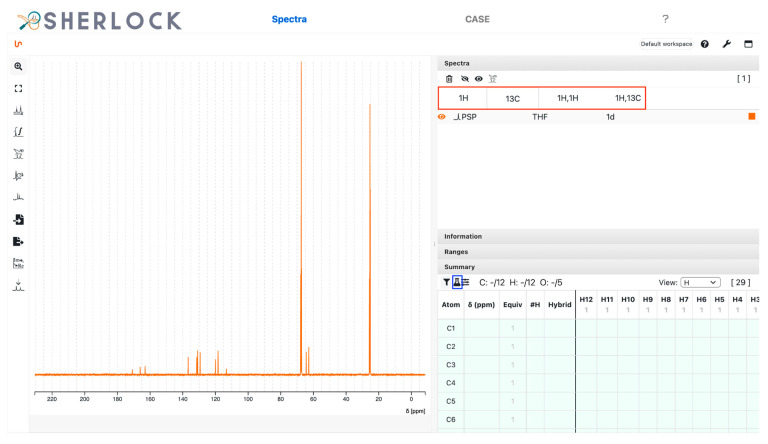
Test compound 15. *Spectra* tab with an overview of imported spectra (red frame) and the summary panel on the right. On the left side is the view of the ^13^C spectrum and a list of tools. The blue framed button is used to set the MF and placeholder atoms.

This section provides a detailed demonstration of the structure elucidation workflow in Sherlock. First, the available NMR spectra (^1^H, ^13^C, HSQC, HMBC, COSY) of compound 15 (Figure 3) were imported into NMRium (*Spectra* tab). Tetrahydrofuran (THF, see Figure 4) was used as a solvent.

The panel in the upper right corner in Figure 4 shows that the spectra were loaded in the frontend (tabs in red frame). The summary panel in the lower right corner contains all the atoms added as placeholders to take into account the user-supplied MF (C_12_H_12_O_5_). The left side panel shows the ^13^C NMR spectrum.

**Figure 5:**
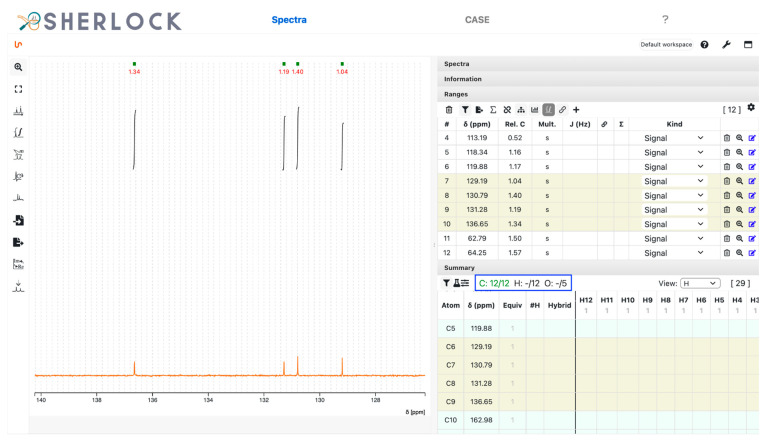
Test compound 15. Zoomed-in ^13^C spectrum with picked ranges and respective entries in the summary panel (row). Ranges of the current spectrum view and the corresponding atoms in the summary table are highlighted.

The summary table was then updated according to the positions of the peaks detected in the 1D and 2D spectra. All twelve ^13^C signals were identified and the counter for carbon atoms in the summary panel (blue frame in Figure 5) appeared green. Solvent signals can be marked as such. The correlation data extraction routines do not consider signals which have been changed to another signal kind than “signal” (default, see Figure 5). In this example, solvent peaks were not picked.

**Figure 6:**
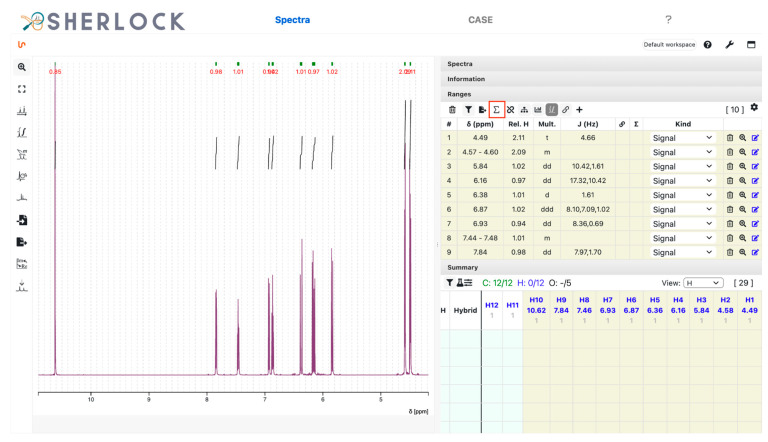
Test compound 15. Zoomed-in ^1^H spectrum with picked chemical shift ranges, peak integrals and the corresponding values in the summary panel (column).

The ^1^H spectrum analysis identified ten chemical shift ranges, each with a single signal and a relative integration value (Figure 6). To set the number of expected protons and to calculate the relative integrals, the button to change the sum of all ranges was used (red framed in Figure 6). Eight ranges had a relative integral value close to one and two of them close to two.

**Figure 7:**
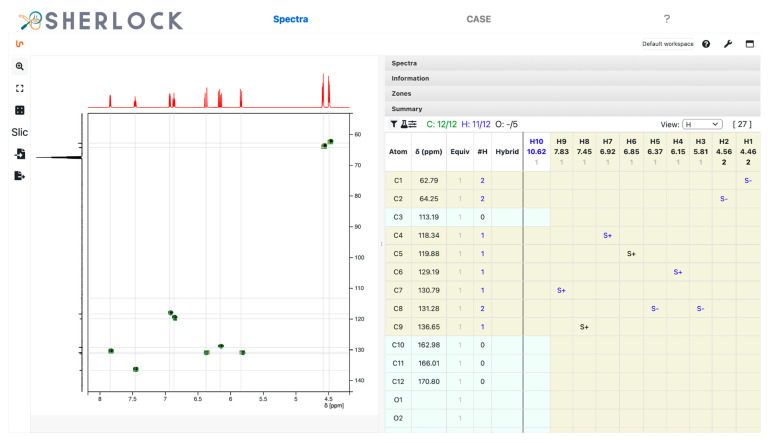
Test compound 15. Multiplicity-edited ^1^H,^13^C HSQC spectrum with indications in the summary table by S+/- and the assigned number of attached protons for carbons.

The HSQC spectrum contains multiplicity-sensitive information (Figure 7). The summary table was filled up with “S+” (single bond, positive peak intensity) or “S-” (single bond, negative peak intensity) during the peak zones picking. Based on this, the system pre-sets the number of attached protons to the matching heavy atom. For positive it will be “1,3” (one or three), which has to be set to one of those values by the user, and for negative the value of 2. In total, 9 signals were picked, two of which belong to the diastereotopic proton pair bound to carbon C8. A proton signal (H10) was left unassigned. The multiplicity of all carbons correlating to a positive HSQC signal was set to 1 since no integration value with close to 3 exists in the ^1^H spectrum.

**Figure 8:**
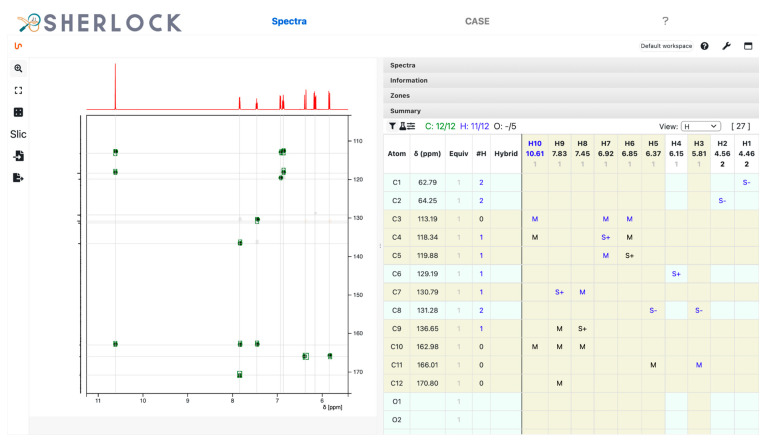
Test compound 15. ^1^H,^13^C HMBC spectrum and the 14 displayed signals reported in the summary table as “M”.

**Figure 9:**
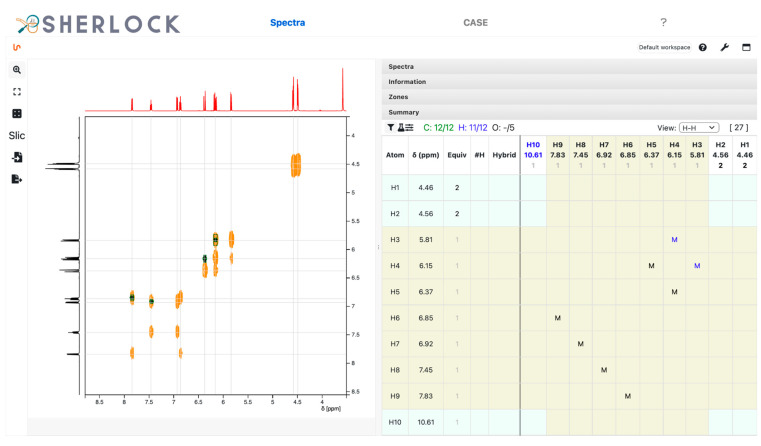
Test compound 15. ^1^H,^1^H COSY spectrum with four entries in the summary table.

The HMBC spectrum and correlated chemical shifts are shown in Figure 8. The “M” in the summary table symbolises the multiple bond correlations established from HMBC signals. In addition, Figure 9 shows the signals in the COSY spectrum.

**Figure 10:**
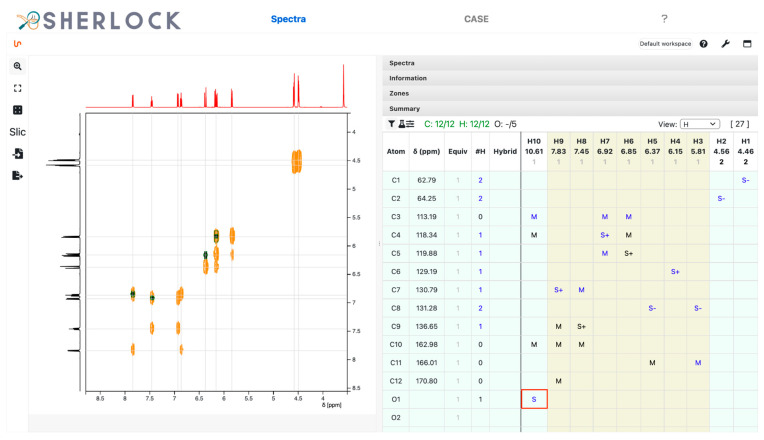
Test compound 15. Assignment of H10 to one of a hydroxyl group. A right click in the cell framed in red and related to row O1 and column H10 opens a context menu to select from.

H10 is still unassigned. Due to the absence of any other heavy atom where it could bind to, this proton can only be bound to one of the oxygens (O1 in this case). This was assigned by a right-click on the corresponding cell (Figure 10). The colour for the displayed number of assigned protons in the panel header changed to green, thus meaning that all hydrogen atoms were bound to a heavy atom. Note that H10 also presents HMBC correlations.

**Figure 11:**
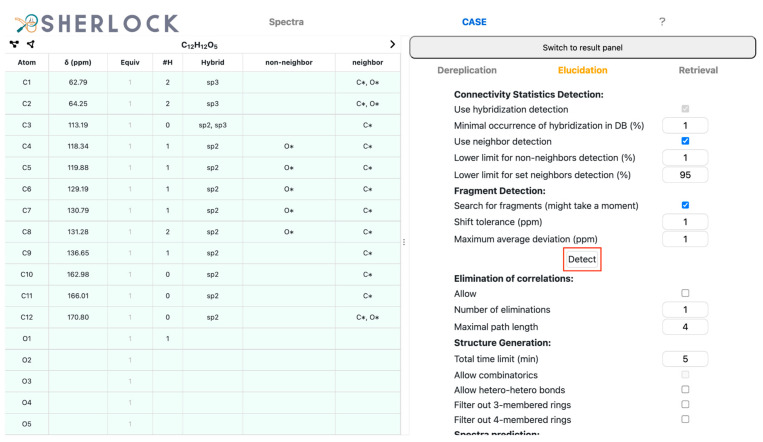
Test compound 15. *CASE* tab with an overview of correlations, detected hybridizations and neighbourhood constraints on the left. The *Query* and *Elucidation* tab is on the right. The button for the detection routine is framed in red.

Subsequently, the *CASE* tab was selected to switch to CASE-related overviews and settings. In the query panel on the right side, the *Elucidation* tab is selected. Clicking the *Detect* button (red framed in Figure 11) started the analysis routines. The results are visible on the left side of the *CASE* tab. The output of the hybridization or neighbourhood detection is shown in the non-neighbour and neighbour columns in the summary table. In the case of atom C3 (113.19 ppm), two hybridizations state proposals were present while this information was unambiguous for the others. That means the frequency rate of these two hybridizations was at least 1% among all occurrences for this requested signal position, multiplicity and proposed MF. The spectral characteristics of atoms C1 (62.79 ppm) and C2 (64.25 ppm) lead to the statistics-based assumption of having at least one carbon and oxygen as neighbours. In addition, oxygen is considered a forbidden neighbour for C4 to C8 (118.34 to 131.28 ppm).

**Figure 12:**
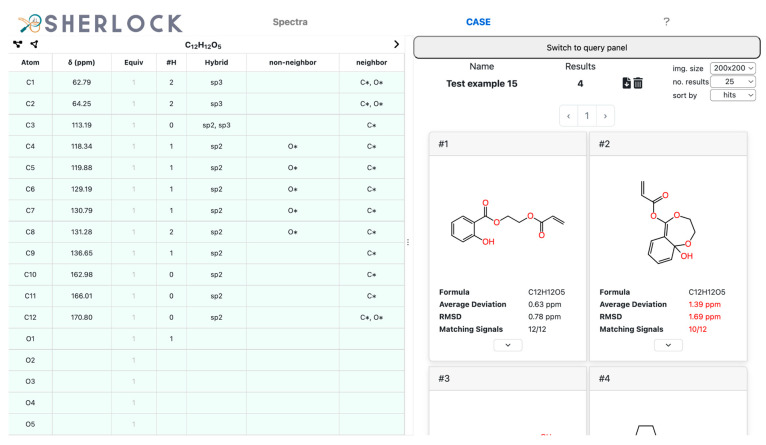
Test compound 15. *CASE* tab with the *Result* tab on the right showing the list of results, the metadata of the elucidation process, and a list of actions, such as the download of the result in an SDF file or the change of image size.

The *Elucidation* button (at the bottom of the *Elucidation* tab, not visible here) started the structure generation process which in this case completed in about one second. Four structures were suggested (Figure 12), with the expected one at the first place in the list. The chemical shifts of all carbons were predicted and the average deviation to experimental values was about 0.63 ppm.

**Figure 13:**
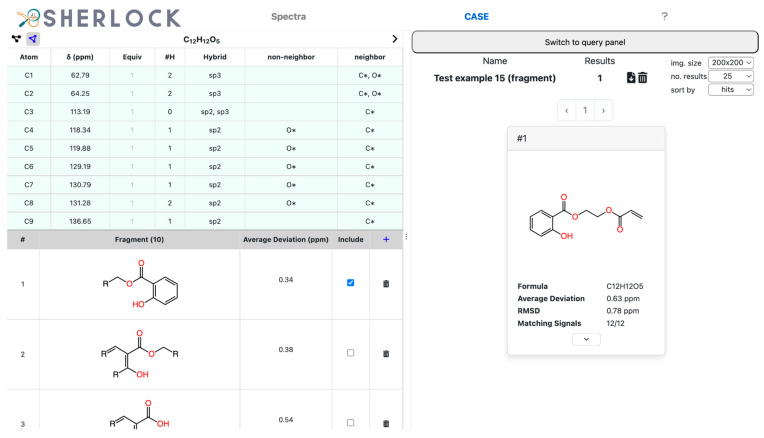
Test compound 15. *CASE* tab with the detected fragments (first one is selected) on the bottom left and the *Result* tab on the right containing the expected molecule only.

No prior knowledge or user influence, such as a manually added or imported fragment, was used in this example, showing that no change of the CASE settings was needed to produce the correct structure. Additionally, the expected structure appeared at the first place in the ranking list. To further narrow down the number of results the fragment search was carried out and the most likely fragment selected for inclusion in the solution structures. Starting the elucidation again left only one candidate in the result list (Figure 13). Thus, the most likely fragment was successfully incorporated in the unambiguous solution of test case 15.

## 3. Methods

**Figure 14:**
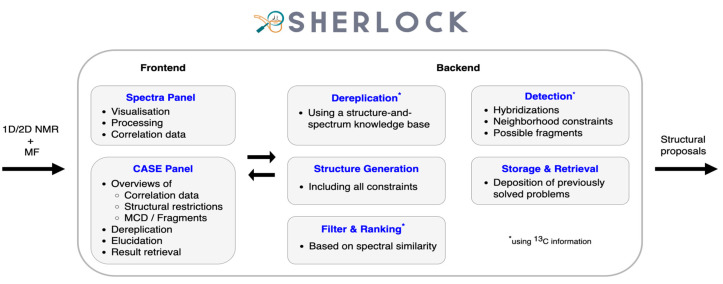
Workflow of the Sherlock software.

The Sherlock CASE software consists of two parts, a frontend and a backend (Figure 14). The frontend acts as a graphical user interface (GUI). It serves for spectra and structure visualisation and for the adjustment of parameters related to NMR data processing and to CASE tasks. The backend runs services that enable database lookup for the identification of known compounds (dereplication) and for the proposal of structural constraints and molecular fragments useful to narrow the chemical search space during elucidation. Tools for ^13^C NMR chemical shift prediction as well as solution structure filtering and ranking complete the set of functions present in CASE programs [2,8]. In addition, the backend also manages the storage and retrieval of previously obtained elucidation results.

Sherlock assumes that 1D and 2D NMR data and the molecular formula of a pure compound are provided as input. In the end, it returns a list of ranked candidate structures that satisfy the constraints expressed by input data.

### 3.1. Structure-and-Spectrum Database Design

Sherlock offers several CASE-related services which rely on its own knowledge base of structure and spectrum assignments [7,8,16,20]. This knowledge base is involved in the dereplication from a list of ^13^C NMR chemical shifts and is used for the construction of a molecular fragments library. The calculation of the probability for a given chemical shift to be related to a particular structural feature, as used for constrained structure generation, also depends on Sherlock’s internal database.

The knowledge base consists of 892.841 records. Each record contains the full description of a molecular structure as a collection of atoms and bonds. Each ^13^C NMR chemical shift value is associated with a multiplicity (the number of attached protons) and an equivalence index. This index indicates the number of chemically equivalent carbon atoms existing in a molecule for a given chemical shift. 

Each newly inserted spectrum is checked during database construction for the existence of signals with the same chemical shift and multiplicity. If identity occurs, then the equivalence descriptor of the concerned atoms is set to the number of such signals. Consequently, the sum of all equivalence indexes of a spectrum is equal to the number of carbons in the currently inserted molecule. 

**Figure 15:**
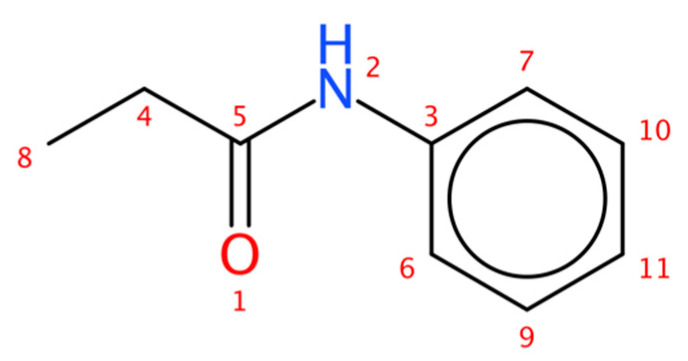
N-Phenylpropanamide (test case 2) with atom numbering.

Figure 15 and Table 2 contain an example where the equivalence index of two signals from a monosubstituted aromatic ring is different from 1. Atoms 6 and 7 as well as 9 and 10 are assigned to a single ^13^C NMR signal in each case.

Each ^13^C spectrum entry in NMRShiftDB [21] was stored in the format presented in Figure 15 and Table 2 to build the reference database. In total 27.938 experimental and predicted spectra were extracted. In addition, the chemical shift verification tool in Advanced Chemistry Development, Inc. (ACD Labs) C+H Predictors and DB software was used to predict the ^13^C spectra of all molecules (864.903) in the natural product database COCONUT [22]. These structures and spectra were subsequently inserted into Sherlock’s knowledge base.

### 3.2. Frontend

#### 3.2.1. *Spectra* Tab

The NMR spectra are displayed and analysed in Sherlock’s frontend by means of the open-source NMR software component NMRium [23]. Spectrum analysis in this context consists of extracting the position of spectral peaks in 1D and 2D spectra in order to establish lists of chemical shift values and of coupling-mediated correlations between them. The full description of the ^13^C query spectrum (Table 2) is the minimum requirement for the dereplication. In the case of an elucidation procedure, a molecular formula is mandatory. The standard proton (^1^H) spectrum is recommended to enhance the upcoming analysis. The multiplicity of the ^13^C NMR signals can be deduced automatically from the complementary DEPT-90 and DEPT-135 spectra. The ^1^H,X-HMQC/HSQC, ^1^H,X-HMBC, (X = ^13^C or, less frequently, ^15^N), and ^1^H,^1^H-COSY spectra constitute the minimum set of 2D NMR data necessary for automatic structure generation. The recording of multiplicity-edited 2D HSQC (me-HSQC) spectra constitutes a good alternative to the time-consuming acquisition of DEPT spectra. Moreover, ^13^C signal multiplicity can be automatically deduced from me-HSQC spectra.

**Figure 16:**
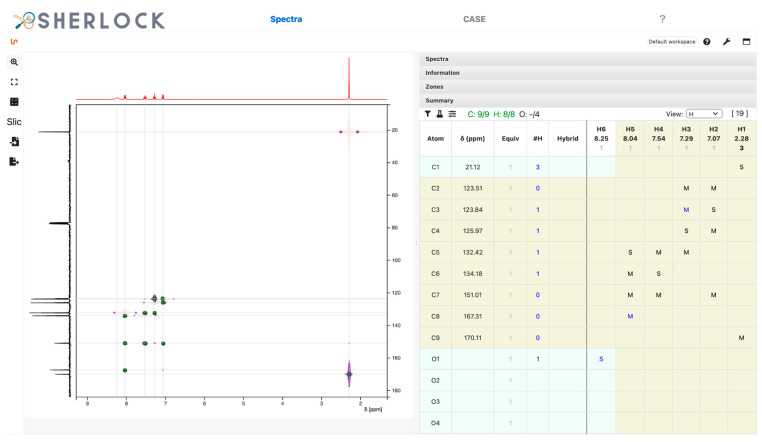
*Spectra* tab with the NMRium component showing superimposed HMQC (in background) and HMBC (in foreground) spectra of test case 32 (aspirin) on the left panel and the summary of the correlations across all 1D and 2D spectra on the right panel.

The analysis of 1D and 2D NMR spectra results in a correlation table (Figure 16). The frontend allows the user to check the validity of spectrum analysis results and to edit it if incorrect data interpretation occurs, before submission to the backend algorithms for dereplication and structure generation.

#### 3.2.2. *CASE* Tab

**Figure 17:**
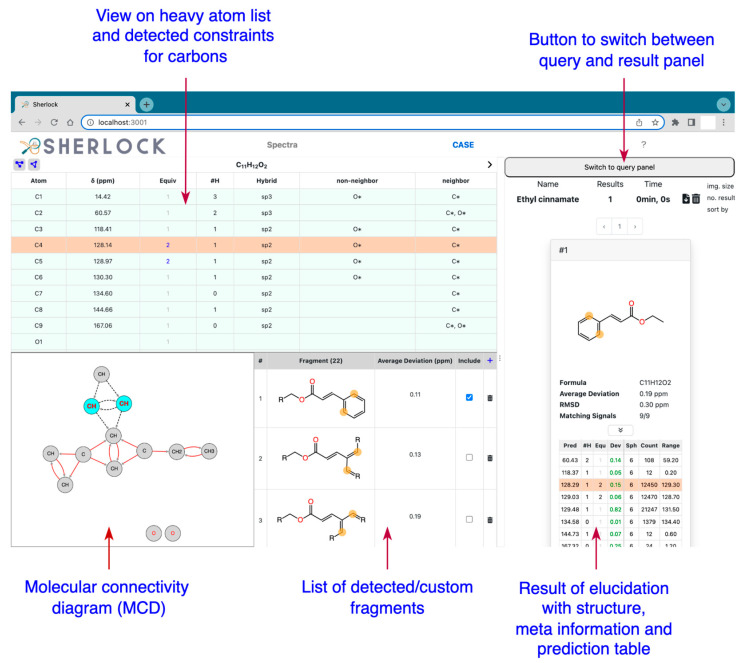
*CASE* tab with different overviews and the result for Ethyl cinnamate (test case 7).

Figure 17 shows the available control panels in the *CASE* tab which assist the user in the elaboration of a spectral data summary and the representation of the molecular connectivity diagram (MCD) [1,8,20,24] derived from 2D NMR correlations. Figure 17 also shows which neighbourhood restrictions for carbons or fragments were automatically detected (Section 3.3.2 and Section 3.3.3) or were manually added by the user. The display component on the right of Figure 17 shows a control panel that allows the user to set specific parameter values needed for dereplication and elucidation. After one of these two tasks is completed the view switches to the result panel which shows the ranked candidate list. Meta information is provided close to structure drawings, in order to help the user to assess the result. Each list of candidate structures produced by an elucidation procedure is stored in Sherlock’s backend and is retrievable at any time.

Further details on how to operate the Sherlock system can be found in the user manual which is embedded in the GUI and thus directly readable in the frontend on the *Info* tab. The user manual is available in the frontend repository (Section 5.1) as well.

### 3.3. Backend

#### 3.3.1. Dereplication

Sherlock supports structural search dereplication, a lookup into a structure-to-spectrum database that prevents any re-elucidation of an already known chemical compound or to retrieve very similar ones [2,4,6,7,8,25]. A structure-to-spectrum knowledge base is necessary for this purpose (see above). The user can select parameters that influence the database screening and the filtering of the result list, such as the tolerance value or maximum allowed average deviation during the chemical shift matching between a ^13^C NMR query spectrum and the ones stored in the database. 

Spectrum comparison relies on a distance calculated for all valid signal pairs. A signal pair is considered valid if the chemical shift matching is successful. Complementary criteria take into account signal multiplicity or equivalence and can be enabled or disabled. 

The closest valid signal pairs are then taken into account for the spectrum-to-spectrum matching. Depending on the number of signals which can be assigned, some of them may be left unpaired because they have no matching counterpart in the other spectrum. Only the matching signal pairs are considered for distance measurement between spectra.

#### 3.3.2. Fragment Library

A list of fragments which should be present in each solution, or goodlist [26,27], can be forwarded to Sherlock. Such substructural information can dramatically shrink the number of possible constitutional isomers after structure generation. Moreover, providing fragments helps to cope with the lack of atom proximity knowledge in problems where proton-poor parts of a molecule exist and thus for which important structural information is unreachable by commonly used heteronuclear 2D NMR experiments HSQC and HMBC. Therefore, fragment data can lead to a noticeable reduction of the candidate list size or give hints to the user who might add complementary structural restrictions manually. [4,5,7,8,24,28]

In order to build a fragment library all entries in the structure-and-spectrum database were used for the fragmentation. Every fragment was created by spherical propagation following the instruction by Elyashberg et al. [8]. Starting at an atom in a molecule, specific conditions preserve connections between heavy atoms are applied to keep important substructural characteristics, such as no bond removal between carbons and hetero atoms or within a ring system if one of the atoms is a starting point [8]. The fragment library consists of around 24.5 million records. Each has a bit string representation to indicate whether a given chemical shift in the assigned subspectrum exists [7,8,28,29]. The database is screened via a bit string comparison during a fragment search where all set bits of a fragment have to be present in the query bitset. Afterwards, every fragment is ranked first by its number of heavy atoms and second by the same spectral matching procedure which is applied during the dereplication (Section 3.3.1).

#### 3.3.3. Statistics-Driven Generation of Structural Constraints

The elucidation process is further supported by the statistical analysis of the structure and spectra database for the determination of complementary structural restrictions. Sherlock is able to detect the likely hybridization states of atoms as well as their forbidden and mandatory atom neighbourhoods.

The previously collected spectral database was used to count what hybridizations or connected atoms for a carbon atom appear. This information is coupled to a tuple consisting of ^13^C chemical shift value, multiplicity and the elemental composition (MF) of a molecule so that for every ^13^C signal in a query spectrum the probability of each hybridization state and of neighbouring atom type can be extracted.

A chemical shift value, a multiplicity and a molecular formula need to be provided in order to request statistical information about hybridization states. In addition, a lower boundary (in percent) is expected to define a minimum occurrence rate for each detected hybridization state compared to all hybridizations stored for a given shift range in the underlying database. If the frequency of a specific hybridization does not reach that given threshold (1% by default), then it will be discarded. 

Similar to the hybridization search, a minimum occurrence (1% by default) of a neighbour atom type is required. Otherwise, such an atom type will be treated as non-neighbor (forbidden) for the carbon atom(s) bearing that ^13^C request signal. If an atom type appears more or equally often compared to the upper boundary (95% by default) it will be considered as a mandatory neighbour. All elements with an occurrence between those two boundaries might or might not be neighbours of an atom during the structure generation. 

Sherlock also checks the frequencies of connections between hetero atoms for a given MF. If the amount of such connections reaches a minimal occurrence of 1% among all connections, then hetero-hetero bonds (HHB) are allowed during structure generation. 

#### 3.3.4. Structure Generation

PyLSD [12,30], a free and open-source powerful software for CASE, takes charge of the structure generation task in the Sherlock backend. It relies on the LSD [11,31] structure elucidation software and provides the ability to deal with atoms with incompletely defined multiplicity or hybridization state. User-defined and automatically detected constraints are passed to pyLSD. Its built-in mechanism of solution ranking was disabled in Sherlock and replaced by a more recent tool (Section 3.3.5).

#### 3.3.5. Spectra Prediction and Ranking

PyLSD-generated candidate structures are ranked according to the similarity of predicted and measured spectra. Prediction relies on a HOSE code-based [32] approach commonly used in CASE systems [2,4,7,8,12,21]. The prediction tool in Sherlock makes use of stereo-enhanced HOSE codes [18].

The number of spheres involved in the creation of the HOSE code library ranges from one to six. During spectra prediction, the HOSE code for the highest number of spheres is created first. If there is no matching entry in the knowledge base, the number of spheres is decreased until matching becomes possible or the number of spheres reaches zero. In the latter case, the prediction for the carbon atom is not possible since no values for a prediction exist. [21]

During the prediction, the number of HOSE code spheres in use, the number of entries as well as chemical shift range are stored to enable a posteriori quality assessment. The final step of the structure elucidation process is to rank the candidate list according to the spectral similarity between the predicted spectra and the experimental one [20]. 

The result (limited to 500 structures) and the CASE-related settings are stored in Sherlock’s backend service to be retrieved at any time.

## 4. Conclusions

An unambiguous interpretation of NMR data can be challenging, due to the many combinatorial possibilities that arise in constitutional space. To address this problem and to support organic chemists in structure determination tasks we introduced Sherlock, a free and open-source software for computer-assisted structure elucidation (CASE). Sherlock’s functionality covers the common steps of structure determination.

It provides the processing and visualisation of NMR data produced in common NMR file formats (Bruker, JEOL, JCAMP-DX). Here, the range of functionality extends from data processing to automatic peak picking up to a summary of all correlations between the different 1D and 2D spectra.

Furthermore, a given molecular formula and the correlation information are used for the dereplication through a spectral knowledge base or the *de novo* elucidation of an unknown compound. The latter includes a lookup for structural constraints for carbon atoms derived by different statistics or fragment search which serve as input for the structure generator and make it possible to further reduce the set of solutions significantly.

Finally, the user receives a list of structure proposals ranked according to the similarity of each predicted spectrum to the experimental one. The results are stored in the system and can be retrieved at any time. The system is able to handle and solve most of the 45 problems used for validation, even with a heavy atom number up to forty.

## 5. Implementation, Software and Test Data

### 5.1. Implementation

The frontend and backend are fully separated software pieces and exchange data only. Hence, they work independently from each other, a feature which enables the general replacement of one of these two components if desired.

Frontend and backend are available from the internet as Docker containers and can either be run locally or deployed on any cloud system that supports Docker. No login functionality is implemented so far. In a publicly accessible server-based solution, any user can access the results and data of others, even on an offline computer with multiple users. A login feature will be implemented in the future to provide data confidentiality to the users.

The backend system supports the storage of atom environments and NMR chemical shift values in the knowledge base (Section 3.1) for a wide range of nuclei types, including ^1^H or ^15^N. Nevertheless, the presently implemented knowledge base contains ^13^C information only and hence dereplication (Section 3.3.1), spectrum prediction and solution ranking (Section 3.3.5) rely solely on ^13^C NMR. The incorporation of NMR data of other nuclei types (e.g., ^1^H, ^15^N) and software developments will be necessary to expand the scope of these operations. Consequently, the *CASE* tab in the frontend will be extended in future works to enable for multiple spectrum-to-spectrum comparisons and the display of their results.

The project descriptions and installation guides are available under https://github.com/michaelwenk/sherlock-frontend (frontend, accessed on 22 November 2022) and https://github.com/michaelwenk/sherlock (backend, accessed on 22 November 2022).

The backend system uses CASEkit (https://github.com/michaelwenk/casekit, accessed on 22 November 2022), a computational library for computer-assisted structure elucidation which is based on Java and the Chemistry Development Kit [33,34].

### 5.2. Software and Test Data

For the processing and results presented in this manuscript, the Digital Object Identifiers (DOI) to the free accessible archived software and complete test data, including the NMRium files used for the CASE purposes, are given in Table 3 and Table 4. The aim is to follow the idea of Research Objects [35] and FAIR data [36] principles. The structures of the test datasets are provided in the supplementary materials.

As mentioned in the introduction and to the best of our knowledge, there is no other free and open-source CASE tool with a similar set of both spectral processing and CASE-related functionalities. Hence, a fair and comprehensive comparison between Sherlock and those CASE tools is not possible due to their commercial nature.

## Figures and Tables

**Table 1:** Overview of the test set used for the validation of Sherlock with planar chemical structures, the number of results regarding the applied constraints and execution time (structure generation, filtering, ranking), the average deviation as well as the number of matching and total signals. Compound names without an asterisk indicate that the dereplication was successful by using the default settings. One asterisk means that parameter adjustments were needed to achieve dereplication, while entries with two asterisks were not found in the knowledge base.

#	Structure	Solutions	Rank	Duration (Sec)	Settings	Average Deviation (ppm)	Matching Signals
**1**	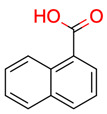 1-Naphthoic Acid	2	1	1	default	0.25	11/11
		1	1	1	first fragment		
**2**	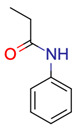 N-Phenylpropanamide	1	1	1	default	0.23	7/7
		1	1	1	first fragment		
**3**	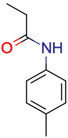 N-(4-Methylphenyl) propanamide	2	1	1	default	0.60	8/8
		1	1	1	first fragment		
**4**	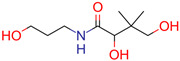 Panthenol	10	1	1	default	0.17	7/9
		1	1	1	first fragment		
**5**	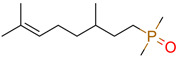 ** 8-(dimethylphosphoryl) -2,6-dimethyloct-2-ene	1	1	1	NN: 0.1% SN: 100%	1.77	11/11
		1	1	1	first fragment		
**6**	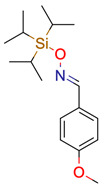 ** 4-Methoxybenzaldehyde O-[tris(1-methylethyl)silyl]oxime	9	1	3	default	1.16	8/8
		2	1	1	first fragment		
**7**	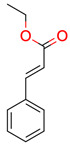 Ethyl cinnamate	2	1	1	default	0.19	9/9
		1	1	1	first fragment		
**8**	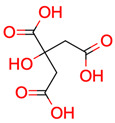 * Citric acid	6	1	1	default	1.90	4/4
		6	1	1	first fragment		
**9**	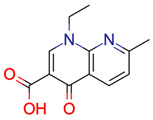 * Nalidixic acid	> 500	1	63	default	1.69	12/12
		-	-	-	first fragment		
		1	1	4	first fragment (DEV: 3 ppm)		
**10**	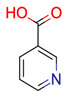 Nicotinic acid	1	1	1	default	2.09	6/6
		1	1	1	first fragment		
**11**	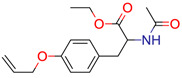 ** Ac-Tyr(allyl)-OEt	9	1	1	default	0.44	14/14
		1	1	1	first fragment		
**12**	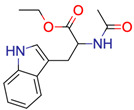 **N-Ac-Trp-OEt	1	1	1	default	0.70	15/15
		1	1	1	first fragment		
**13**	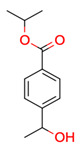 ** propan-2-yl 4-(1-hydroxyethyl)benzoate	2	1	1	default	1.32	9/9
		1	1	1	first fragment		
**14**	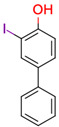 ** 2-iodo-4-phenylphenol	2	1	1	default	0.90	10/10
		-	-	-	first fragment		
**15**	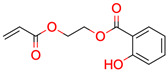 ** 2-prop-2-enoyloxyethyl 2-hydroxybenzoate	4	1	1	default	0.63	12/12
		1	1	1	first fragment		
**16**	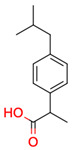 Ibuprofen	1	1	1	default	0.35	10/10
		1	1	1	first fragment		
**17**	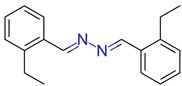 ** 1,2-Bis [(*o*-ethylphenyl) methylene]hydrazine	2	1	380	HHB	1.72	7/9
		-	-	-	first fragment		
**18**	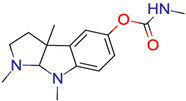 Eserine	6	1	1	default	0.28	15/15
		1	1	1	first fragment		
**19**	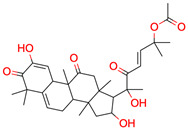 Cucurbitacin E	4	1	2	default	0.66	28/32
		1	1	1	first fragment		
**20**	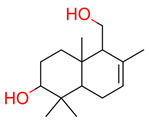 3-hydroxy-drimenol	1	1	1	default	1.36	13/15
		1	1	1	first fragment		
**21**	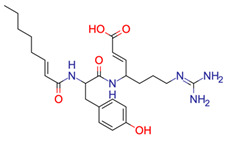 ** Barnesin A	133	8	116	default	1.92	23/23
		11	4	109	first fragment		
**22**	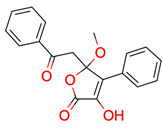 Allantofuranone	336	1	28	default	0.11	15/15
		1	1	2	first fragment		
**23**	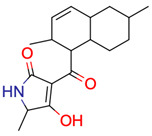 ** 3-[(2,6-dimethyl-1,2,4*a*,5,6,7,8,8*a*-octahydronaphthalen-1-yl)-hydroxymethylidene]-5-methylpyrrolidine-2,4-dione	273	6	25	default	2.12	17/18
		273	6	19	first fragment		
**24**	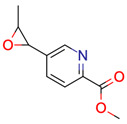 Caripyrin	5	1	1	default	0.74	10/10
		1	1	1	first fragment		
**25**	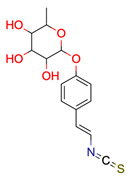 ** Sinapigladioside	> 500	1	705	HYBR: 0.1% SN: 100%	1.02	12/13
		13	1	18	first fragment		
**26**	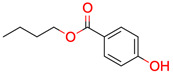 Butylparaben	1	1	1	default	0.33	9/9
		1	1	1	first fragment		
**27**	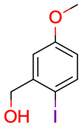 (2-iodo-5-methoxyphenyl)methanol	2	1	1	default	0.03	8/8
		1	1	1	first fragment		
**28**	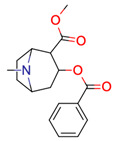 Cocaine	94	1	8	default	0.95	15/15
		1	1	1	first fragment		
**29**	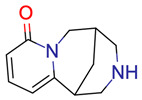 Cytisine	3	1	1	default	0.41	11/11
		1	1	1	first fragment		
**30**	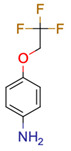 ** 4-(2,2,2-trifluoroethoxy)aniline	3	1	1	default	1.21	5/6
		-	-	-	first fragment		
		1	1	1	first fragment (DEV: 2 ppm)		
**31**	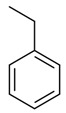 Ethylbenzene	2	1	1	default	0.51	6/6
		1	1	1	first fragment		
**32**	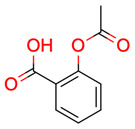 Aspirin	8	1	1	default	0.50	9/9
		1	1	1	first fragment		
**33**	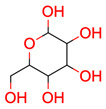 α-D-glucose	2	1	1	default	0.40	6/6
		1	1	1	first fragment		
**34**	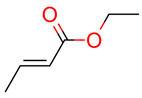 Ethyl but-2-enoate	1	1	1	default	0.34	6/6
		1	1	1	first fragment		
**35**	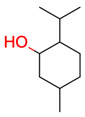 Menthol	3	1	1	default	0.94	8/10
		1	1	1	first fragment		
**36**	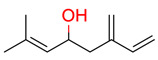 (4R)-Ipsdienol	1	1	1	default	0.78	10/10
		1	1	1	first fragment		
**37**	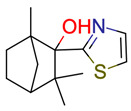 ** 1,3,3-trimethyl-2-(1,3-thiazol-2-yl)bicyclo [2.2.1]heptan-2-ol	301	1	18	default	1.63	10/13
		-	-	-	first fragment		
		26	1	2	first fragment (DEV: 4 ppm, AVGDEV: 2 ppm)		
**38**	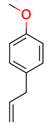 Estragole	1	1	1	default	0.18	8/8
		1	1	1	first fragment		
**39**	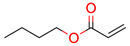 Butyl acrylate	1	1	1	default	0.57	7/7
		1	1	1	first fragment		
**40**	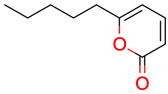 6-Pentyl-2H-pyran-2-one	14	1	1	default	0.12	10/10
		1	1	1	first fragment		
**41**	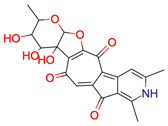 ** Rubterolone A	No result					
**42**	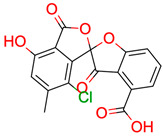 ** Actinospirol A	No result					
**43**	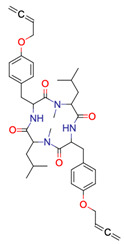 ** Pseudoxylallemycin B	No result					
**44**	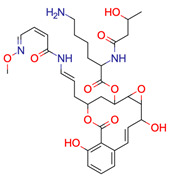 ** Necroxime A	No result					
**45**	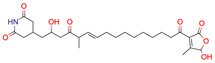 ** Gladiofungin A	No result					

**Table 2:** Carbon atoms in N-Phenylpropanamide (test case 2) and their assigned spectral properties.

Atom	^13^C Signal		
Index	Chemical Shift (ppm)	Equivalence	Number of Protons
3	137.9	1	0
4	30.8	1	2
5	172.2	1	0
6,7	119.8	2	1
8	9.89	1	3
9,10	129.0	2	1
11	124.2	1	1

**Table 3:** Overview and DOIs belonging to software archives used in frontend and backend services of Sherlock.

Description	DOI
Frontend (source code, v1.0.4)	https://doi.org/10.5281/zenodo.7032805
Frontend (Docker image, v1.0.4)	https://doi.org/10.5281/zenodo.7032810
Backend (source code, v1.1.0)	https://doi.org/10.5281/zenodo.7037546
Backend (Docker images, v1.1.1)	https://doi.org/10.5281/zenodo.7115924
CASEkit (source code, v1.0.1)	https://doi.org/10.5281/zenodo.7115819

**Table 4:** Overview and DOIs to datasets applied for validation of Sherlock.

Test Case	DOI
1	https://doi.org/10.57992/NMRXIV.P10.S69
2	https://doi.org/10.57992/NMRXIV.P10.S62
3	https://doi.org/10.57992/NMRXIV.P10.S61
4	https://doi.org/10.57992/NMRXIV.P10.S60
5	https://doi.org/10.57992/NMRXIV.P10.S55
6	https://doi.org/10.57992/NMRXIV.P12.S79
7	https://doi.org/10.57992/NMRXIV.P12.S75
8	https://doi.org/10.57992/NMRXIV.P12.S76
9	https://doi.org/10.57992/NMRXIV.P12.S74
10	https://doi.org/10.57992/NMRXIV.P12.S73
11	https://doi.org/10.57992/NMRXIV.P12.S77
12	https://doi.org/10.57992/NMRXIV.P12.S78
13	https://doi.org/10.57992/NMRXIV.P9.S53
14	https://doi.org/10.57992/NMRXIV.P9.S52
15	https://doi.org/10.57992/NMRXIV.P9.S54
16	https://doi.org/10.57992/NMRXIV.P9.S51
17	https://doi.org/10.57992/NMRXIV.P9.S50
18	https://doi.org/10.57992/NMRXIV.P8.S48
19	https://doi.org/10.57992/NMRXIV.P8.S47
20	https://doi.org/10.57992/NMRXIV.P6.S42
21	https://doi.org/10.57992/NMRXIV.P6.S41
22	https://doi.org/10.57992/NMRXIV.P11.S72
23	https://doi.org/10.57992/NMRXIV.P11.S71
24	https://doi.org/10.57992/NMRXIV.P7.S46
25	https://doi.org/10.57992/NMRXIV.P5.S38
26	https://doi.org/10.57992/NMRXIV.P1.S2
27	https://doi.org/10.57992/NMRXIV.P1.S1
28	https://doi.org/10.57992/NMRXIV.P10.S57
29	https://doi.org/10.57992/NMRXIV.P10.S68
30	https://doi.org/10.57992/NMRXIV.P10.S67
31	https://doi.org/10.57992/NMRXIV.P4.S37
32	https://doi.org/10.57992/NMRXIV.P10.S56
33	https://doi.org/10.57992/NMRXIV.P10.S58
34	https://doi.org/10.57992/NMRXIV.P10.S63
35	https://doi.org/10.57992/NMRXIV.P10.S59
36	https://doi.org/10.57992/NMRXIV.P10.S70
37	https://doi.org/10.57992/NMRXIV.P10.S65
38	https://doi.org/10.57992/NMRXIV.P10.S66
39	https://doi.org/10.57992/NMRXIV.P10.S64
40	https://doi.org/10.57992/NMRXIV.P9.S49
41	https://doi.org/10.57992/NMRXIV.P6.S44
42	https://doi.org/10.57992/NMRXIV.P6.S45
43	https://doi.org/10.57992/NMRXIV.P6.S43
44	https://doi.org/10.57992/NMRXIV.P5.S40
45	https://doi.org/10.57992/NMRXIV.P5.S39

## Data Availability

Software and the test dataset is freely available (see Section 5.2).

## Supplementary Materials

The following supporting information can be downloaded at: https://www.mdpi.com/article/10.3390/molecules28031448/s1, The forty-five structures of the test dataset are available as SMILES.

## Abbreviations

NMR	Nuclear Magnetic Resonance
CASE	Computer-Assisted Structure Elucidation
MS	Mass Spectrometry
GUI	Graphical User Interface
MF	Molecular Formula
ppm	Parts Per Million
MCD	Molecular Connectivity Diagram
NN	Non-Neighbor (forbidden)
SN	Set Neighbour (mandatory)
HYBR	Hybridization
HHB	Hetero-Hetero Bond
DEV	Allowed Deviation (tolerance)
AVGDEV	Allowed Average Deviation
RMSD	Root-Mean-Square Deviation
HSQC	Heteronuclear Single Quantum Coherence
me-HSQC	Multiplicity-edited HSQC
HMBC	Heteronuclear Multiple Bond Correlation
COSY	Correlated Spectroscopy
DEPT	Distortionless Enhancement by Polarisation Transfer
THF	Tetrahydrofuran
DOI	Digital Object Identifier
